# A Fuzzy-Based Approach for Sensing, Coding and Transmission Configuration of Visual Sensors in Smart City Applications

**DOI:** 10.3390/s17010093

**Published:** 2017-01-05

**Authors:** Daniel G. Costa, Mario Collotta, Giovanni Pau, Cristian Duran-Faundez

**Affiliations:** 1 Department of Technology, State University of Feira de Santana, Feira de Santana 44036-900, Brazil; 2 Faculty of Engineering and Architecture, Kore University of Enna, Enna 94100, Italy; mario.collotta@unikore.it (M.C.); giovanni.pau@unikore.it (G.P.); 3 Department of Electrical and Electronic Engineering, University of the Bío-Bío, Bío Bío Region 4051381, Chile; crduran@ubiobio.cl

**Keywords:** smart cities, visual monitoring, fuzzy-based configuration, visual sensor networks

## Abstract

The advance of technologies in several areas has allowed the development of smart city applications, which can improve the way of life in modern cities. When employing visual sensors in that scenario, still images and video streams may be retrieved from monitored areas, potentially providing valuable data for many applications. Actually, visual sensor networks may need to be highly dynamic, reflecting the changing of parameters in smart cities. In this context, characteristics of visual sensors and conditions of the monitored environment, as well as the status of other concurrent monitoring systems, may affect how visual sensors collect, encode and transmit information. This paper proposes a fuzzy-based approach to dynamically configure the way visual sensors will operate concerning sensing, coding and transmission patterns, exploiting different types of reference parameters. This innovative approach can be considered as the basis for multi-systems smart city applications based on visual monitoring, potentially bringing significant results for this research field.

## 1. Introduction

Modern cities have several issues regarding resources management, security, urban mobility, disaster recovery, among many others, which can be supported by sensors. Actually, different kinds of sensing technologies can be used to help city management by automating some tasks and predicting problems before they occur [1,2]. In this context, Wireless Sensor Networks (WSN) can be used to create a smart city infrastructure, as sensors can monitor different data, e.g., water pressure, noise, luminance, electric current, traffic, among others, activating alarms or automated systems in the occurrence of some events [3,4].

For a lot of monitoring applications in a smart city environment, visual sensors can provide valuable information of monitored areas. Actually, still images and video streams can be processed for different tasks [5,6]: distributed or centralized processing of visual data, usually centered at pattern recognition, has already been exploited for many applications in urban contexts, as in parking management and traffic control. The use of visual sensing can then create a promising scenario for the adoption of Wireless Visual Sensor Networks (WVSNs), but many challenging issues are raised when visual sensors are deployed [7], demanding proper solutions.

Smart city monitoring employing cameras is a relevant research topic that has fostered the development of many research works in last years. In [8], authors proposed an approach to use cameras and sensor nodes in order to provide an efficient surveillance system, calculating the correct position of events of interest for camera rotation. In [6], cameras are used to monitor movement of targets for public security. The work in [9] employs visual sensors to manage parking lot occupancy. Actually, many such systems could be merged to provide integrated services in smart cities, but efficient configuration of visual sensors is still a relevant issue that should be optimized for higher efficiency.

As there may be many concurrent wireless sensor networks operating in a smart city scenario, efficient configuration of sensor nodes is of paramount importance. Therefore, a relevant issue of wireless visual sensor networks is the proper configuration of sensor nodes, as visual data transmission may be too degrading in terms of energy consumption and transmission throughput. In this work, we define *configuration* as beng centered in three different elements: sensing, coding and transmission. These elements are defined as follows:

- *Sensing*: it indicates the sensing behavior of visual sensors. For cameras retrieving still images, it may define the sampling frequency, which reflects in the number of snapshots taken per second. For video monitoring, the sensing behavior may indicate transmission bursts or continuous streaming.
- *Coding*: source nodes may apply different coding algorithms, with diverse compression ratios and processing costs. Visual data resolution and color patterns are also relevant coding configurations.
- *Transmission*: visual data may be transmitted in real-time, or transmission latency and jitter may not be a concern. Quality of Service (QoS) policies may also be employed over some traffic, which may be prioritized during transmission.

In smart cities, which may be highly dynamic [10], sensor node configurations should regard different parameters from different perspectives and with different significances. In this context, we propose herein a fuzzy-based mechanism to dynamically configure visual sensors, computing and assigning their sensing behavior, coding schemes and transmission approaches according to a series of parameters associated with visual monitoring in smart cities. This fuzzy-based solution takes internal and external parameters to define a unified mechanism to configure the way visual sensors will operate, which is represented as the Sensing/Coding/Transmission Pattern (SCTP). A proposed Fuzzy Logical Controller (FLC) was implemented on a prototyping board and numerical results of SCTP computations are evaluated for different configuration parameters.

Therefore, exploiting fuzzy logic, we expect to provide a generic mechanism to configure visual sensors in any smart city application, requiring only proper definition of the parameters that best represent the monitoring requirements of the considered applications. To the best of our knowledge, such an approach has not been proposed before.

The remainder of this paper is organized as follows. Section 2 presents some related works. Section 3 brings the definitions of visual monitoring in WVSNs. The proposed approach is defined in Section 4. Numerical results are presented in Section 5, followed by conclusions and references.

## 2. Related Works

Smart city monitoring is a relevant research topic that has fostered the development of many research works in last years. Actually, some of them influence our investigation in different ways, mostly on visual sensing, QoS provisioning, sensor configuration and Internet of Things (IoT) applications.

Many works have been done on smart city monitoring. In [1], authors have developed a smart city monitoring system to detect leakages and failures in a pipeline infrastructure. Based on wireless sensor networks technology, their system is able to detect, to localize and to quantify anomalies in a water supply system. In a different way, the paper in [11] describes a system to help evacuation of a building, determining the best exit route at each part of the building according to the number of evacuees. An application for Structural Health Monitoring (SHM) in smart cities is presented in [12], where a total of 64 nodes are used to monitor vibrations in the Golden Gate Bridge, in the city of San Francisco.

Surveillance applications in smart cities can also benefit from the use of cameras [7]. In [8], authors proposed an approach to use cameras and sensor nodes in order to provide an efficient surveillance system. In their proposal, several sensor nodes are uniformly deployed on an area of interest, and a remote sink uses the collected data from the sensors to calculate the correct position of an event. Then, by having the position of an event, the sink sends rotation commands to cameras near the source of the event so that they can record what caused it. In [6], cameras are used to monitor movement of targets for public security, with many practical applications. Distributed processing of visual data is proposed in [9], when visual sensors are employed to automatically detect cars in parking areas, which can support efficient parking systems in smart cities. The work in [13] employs cameras and laser beams to monitor structural displacements. In fact, when employing cameras, relevant visual information can be retrieved, potentially enriching smart city applications.

As there are many challenges to building smart cities, research efforts have addressed different aspects of this environment. However, a recurrent relevant issue is to determine how visual sensors will behave in terms of sensing, coding and transmission patterns for each of these challenges. Different optimization approaches have been proposed in last years, addressing such configuration problems in different ways (e.g., addressing only sensing or combined coding and transmission), which is actually more critical for visual sensors when compared to scalar sensors due to more configuration options. As a result, context-aware wireless visual sensor networks have been considered as a feasible option for diverse monitoring functions, and many works have contributed to this area.

Optimized transmission in WVSNs allows the dynamic adjustment of the transmission rate, for example due to congestion in relay nodes or to save energy [14]. The work in [15] proposes dynamic adjustment of the transmission rate in order to save energy. In addition, transmission rate adjustment to face congestion is proposed in [16]. For these approaches, transmission rate is adjusted for higher efficiency, but usually only information of source and relaying nodes is taken when optimizing the network operation, which may not be efficient in smart city scenarios.

A promising approach to change the sensing and transmission behavior of sensor nodes was proposed in [17]. The idea in that work is to define the transmission behavior of visual sensors according to their relevance for the entire network, which may be associated, for instance, with the monitoring of critical events. Similarly, the work in [18] employs scalar sensors to rapidly detect critical events, which are then reflected in higher priority to nearby visual sensors. In both works, the sensing, coding and transmission behavior of visual sensors are established according to the priority of source nodes, but external parameters of smart cities are not still considered.

Actually, many previous works have proposed node configuration following some optimization parameter. We define that most of those works have considered at least one of five different parameters to perform configurations: events, media type, node’s status, data content and network QoS. Table 1 presents some examples of optimization approaches based on some of these parameters.

The configuration problem following optimization parameters is indeed a good approach to optimize sensing, coding or transmission patterns of source nodes, although most works are concerned in achieving some specific performance enhancement. While some works are concerned with performance parameters, as energy consumption and QoS-based transmission improvements [26], there is also a need to change the configuration of sensors to attend different application requirements. The work in [27] proposes a framework to reconfigure wireless sensor networks when heterogeneous applications are considered. The idea is to allow different applications to use the same WSN at different times, without requiring redeployment or use of additional sensors. Such reconfiguration, however, requires not only modification of sensing, coding and transmission patterns of sensors, but also changes in the employed protocol stack, which adds additional challenges. In [28], context-aware wireless sensor networks are also investigated. In this work, a task allocation scheme is proposed for heterogeneous applications using the same network infrastructure, with nodes being allocated to the execution of tasks. Additionally, authors in [29] propose a common programing interface to be used for heterogeneous applications when reconfiguration is performed.

Configuration of sensor nodes may then have different perspectives. Optimal configurations of source nodes for performance enhancement may be highly beneficial, as well as the adoption of reconfiguration frameworks to adapt the network to different application requirements. Although such approaches achieve different levels of sensing, coding and transmission configurations in wireless visual sensor networks, they are not necessarily adequate for smart city scenarios composed of multiple concurrent systems. Moreover, they do not consider external parameters of networks, which are relevant to the dynamics of multi-systems smart cities. This is, in fact, the research gap that is being addressed by this article.

Thus, in a different way compared to previous works, it is proposed herein a fuzzy-based approach aimed at the establishment of the sensing, the coding and the transmission behavior of any number of visual sensors according to different parameters related to the operation of visual sensor networks in smart city environments, providing a broader solution that can be used as a reference for future developments in this area.

## 3. Visual Sensing Configuration

A visual sensor will be typically configured to perform a determined task according to the definitions of an application. For example, a visual sensor may stream video when triggered by a scalar (auxiliary) sensor, or it may continuously transmit image snapshots at some rate. Whatever the case, visual sensors may operate differently according to characteristics of the considered application, which may define sampling frequencies, coding algorithms and transmission patterns for source nodes.

In order to achieve high performance, the operation of visual sensors may be optimized, changing their configurations in some well-defined cases. For example, if energy supply runs low, lower quality visual data may be transmitted by some or all sensors, potentially saving energy. In a different perspective, the occurrence of an event of interest, such as an explosion or traffic congestion, may trigger prioritized transmission from sensors in the vicinity of the event, with tagged packets receiving some QoS guarantees as they are transmitted over the network. Although such configuration approaches may perform well when a single wireless visual sensor network is considered [30], visual sensors may be operating in dissonance with a smart city environment, especially when multiple distinct visual monitoring systems are running concurrently.

When using visual sensors, some applications may consider sensors’ orientations as an important configuration element. If rotatable cameras are employed, sensing directions may be optimized and many works have been concerned with this issue [31,32]. Although we do not consider sensors’ orientations as a configuration element in our fuzzy-based configuration approach, the proposed solution can be combined with algorithms that alter sensors’ orientations. In other words, even if coverage optimization algorithms are employed, sensing, coding and transmission configurations can still be efficiently established using our proposed approach.

Different parameters may impact the overall operation of smart city systems. And although their influence may be neglected in some cases, visual sensing, coding and transmission may be adapted to such parameters, aiming at a more optimal operation of wireless visual sensor networks. Actually, we define that there are two different types of parameters in typical smart city systems, which can influence the operation of visual sensors: *internal* and *external*. Internal parameters are produced and have their influence directly inside the scope of a wireless visual sensor network, and thus they may be inferred from the considered network. On the other hand, external parameters are collected from outside the network and they usually will affect all systems in a smart city scenario.

The next subsections will discuss these two types of parameters, which will be considered in the proposed approach.

### 3.1. Internal Parameters

Much data related to the operation of wireless visual sensor networks can be computed as optimization parameters for configuration purposes. Actually, these parameters should be properly accounted for if optimal configuration is desired, and thus the proposed approach presented in the next section takes a handful of internal parameters.

There may be many internal parameters, with different impacts on the operation of visual sensors. The proper choosing of those parameters is an important design issue, but we expect that some of them will be present in most cases. Those parameters are listed as follows:

- **Camera hardware**: sensor nodes may be equipped with different types of cameras, which may have different hardware characteristics. Cameras with zooming and rotation capabilities may need to transmit more information for some applications. Lens quality and supported image resolutions are also important parameters that may impact sensor operation.
- **Processing power**: processing and memory capabilities will determine which multimedia compression algorithms may be executed, affecting sensing quality and energy consumption over the network. This parameter can then guide the configuration of data coding in sensor nodes.
- **Event-based prioritization**: visual sensors may have different priorities depending on events monitoring [17,30]. Network services and protocols may consider event-based priorities for optimized transmissions. Moreover, most relevant nodes may transmit more data than less relevant sensors, depending on the configurations of the considered monitoring application.
- **Residual energy**: sensor nodes may operate using batteries, which provide finite energy. Therefore, the current energy level of sensor nodes may interfere in the way sensors will retrieve visual information. For example, the sensing frequency of sensors nodes may be reduced when their energy level is below a threshold.
- **Security**: some security concerns may be exploited to differentiate sensor nodes. As an example, regions with confidentiality requirements may demand the use of robust cryptography algorithms [33], which depends on available processing power and efficient energy management.

### 3.2. External Parameters

In a smart city scenario, visual sensor networks should be also configured exploiting external parameters. In general, we define external parameters as all data that are equally significant for all wireless sensor networks in a smart city. Although wireless sensor networks usually do not consider those parameters, monitoring and control systems in smart cities may benefit from the exploitation of such data.

We initially selected four relevant external parameters for optimizations, described as follows:

- **Luminance**: some visual sensors may be able to retrieve visual information during the night or in dark places, but it may not be true for other sensors. The luminance intensity (measured in *lux*) can be considered when defining the sensing frequency of visual sensors.
- **Deployment area**: depending on the considered application and deployment area, visual sensors may need to transmit more visual data. For instance, in public security applications, visual sensors deployed in areas with high levels of criminality may be required to transmit more data than if they were deployed in other areas (even in the absence of events of interest). As a remark, the deployment area is a parameter that has significance for a scenario that may comprise many different WVSNs, which is different than the (internal) prioritization parameter, whose significance is valid for the considered sensor network.
- **Day of the monitoring**: a city is a complex and dynamic environment, where some patterns may be, sometimes, defined. For example, traffic is affected by the day of the week, since fewer cars may be moving around on weekends, or even on holidays. Thus, depending on the application, visual monitoring may be influenced by the day of the monitoring.
- **Relevance of the system**: in a smart city scenario, some systems may be more relevant than others. If we have multiple deployed wireless visual sensor networks, a pre-configured relevance level for the different network operations may be considered as an external parameter, impacting the configuration of the sensor nodes.

## 4. Proposed Approach

In general, visual source nodes may be expected to transmit data in one of three different ways: query-based, triggered-based or time-based [14]. Query-based transmissions are produced by specific queries (usually) from outside the network. For autonomous systems as generally deployed for smart city applications, source nodes will typically transmit information following a triggered-based (in response to some event) or time-based (in response to some schedule) pattern. For these transmission patterns, proper configuration of visual sensors is highly desired, which should be able to be performed dynamically along the time even in the presence of multiple concurrent wireless visual sensor networks.

**Figure 1:**
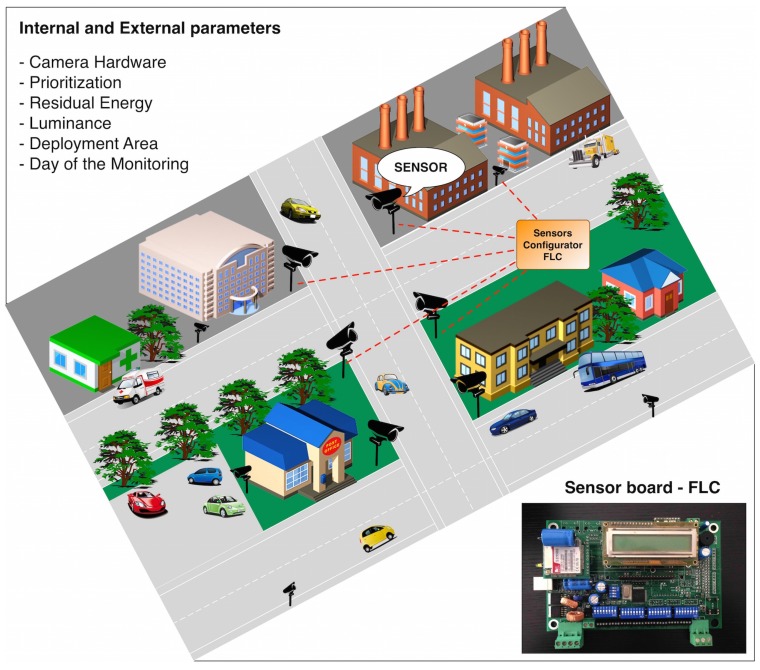
A generic smart city employing the proposed approach.

We propose a Fuzzy Logic Controller (FLC) that will consider some internal and external parameters directly or indirectly inferred from a smart city scenario in order to define the sensing, the coding and the transmission configuration of any number of visual sensors. A representation of a generic smart city adopting the proposed approach is presented in Figure 1.

### 4.1. Fuzzy Logic Controller

The soft computing techniques fit themselves well in wireless visual sensor networks applications, since they have been proposed for the construction of new generation artificial intelligence (high machine intelligence quotient, human-like information processing) and for solving non-linear and mathematically unmodeled systems. Soft computing techniques can be appropriate for several engineering problems, especially for complex problems, where classical control methods do not achieve comparatively favorable results. In addition, it is useful to note that soft computing techniques can be implemented at low cost. FLCs belong to the soft computing techniques that aim to adapt to the pervasive imprecision of the real world and to obtain robust and low cost solutions.

Actually, the use of rule-based Fuzzy Logic Controllers enables the implementation of multi-criteria control strategies [34]. In fact, fuzzy logic is capable of making real-time decisions, even with incomplete information [4]. Conventional control systems rely on an accurate representation of the environment, which generally does not exist in reality. Since fuzzy logic systems can manipulate the linguistic rules in a natural way, they are particularly suitable in several contexts, such as WVSN applications. Moreover, fuzzy systems can be used by blending different parameters and rules that, combined together, may produce an optimal result. In general, an accurate computation may be too complex and it could also be meaningless due to the quick change of the network conditions. For this reason, the FLCs, based on linguistic rules instead of inflexible reasoning, can be the right choice to describe a sampling, coding and transmission pattern in visual sensor networks. Therefore, fuzzy logic has been adopted because it can deal with uncertain and vague values [35,36,37].

As an important remark, FLC does not usually require high computing power to operate, which is a desired characteristic in WSNs when sensor nodes are battery powered. In addition, many works have applied FLCs in order to prolong the battery life on sensor nodes or to manage the application in the reference scenario [38,39,40,41]. Therefore, for the proposed approach, the use of an FLC to compute and assign the SCTP for the sensors should not add significant processing and energy costs.

### 4.2. Configuring the FLC

Although there may be many possible parameters for the FLC, we will define a set of reference parameters, which will be considered for the proposed approach, but any other parameter could be easily exploited in future implementations. The chosen parameters may be directly or indirectly inferred by the FLC, which may communicate with source nodes or be previously configured during deployment.

**Figure 2:**
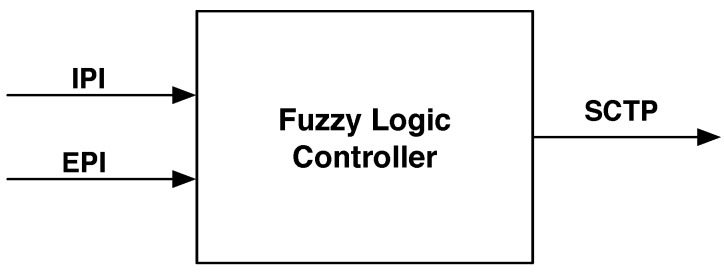
General scheme of the proposed fuzzy-based computation approach.

Considering the internal and external parameters shown previously, it would not be appropriate to develop an FLC having six/seven fixed input parameters. Moreover, this would imply the use of a fairly large number of membership functions. To this end, in this paper, we have chosen to implement an FLC that does not depend on a specific application, and, as a consequence, we are introducing a generic solution for any number of configuration parameters. Actually, it will be possible to regulate the FLC based on specific application fields. In doing so, the proposed FLC is defined as having two types of input parameters, referred to as Internal Parameters Indicator (IPI) and External Parameters Indicator (EPI), respectively. Figure 2 presents a generic scheme of the proposed FLC-based computation of sensors’ configurations. The output of the FLC is the SCTP for visual sensors.

Specifically, these parameters are calculated as a correlation between the internal and external parameters. The IPI and EPI indicators depend on the relevance that each Internal Parameter ($IP_{i}$) and External Parameter ($EP_{i}$) covers for obtaining acceptable performance in any specific application field. Therefore, Table 2 shows the values of an internal or external parameter on the basis of performance requirements defined by the application, which are the considered thresholds. In order to explain better the idea, Table 2 shows a possible representation of these numerical values. The ranges considered in the first column, the corresponding thresholds and the percentage values associated with them, can vary in number and in value depending on the type of application.

The IPI and EPI indicators are calculated as follows:

(1) $$IPI=\sum_{i=1}^{n}\left(a_{i}*IP_{i}\right)\leq 1,$$

(2) $$EPI=\sum_{i=1}^{m}\left(b_{i}*EP_{i}\right)\leq 1.$$

In Equations (1) and (2), *n* is the number of internal and *m* is the number of external parameters, and $a_{i}$ and $b_{i}$ are the relevance of the $IP_{i}$ or $EP_{i}$ parameter in the referred application field, respectively. In detail, $a_{i}$ and $b_{i}$ are dimensionless parameters and behave like a priority, whose minimum value is 0 and the maximum is 1. Moreover, ($a_{i}+a_{\left(i+1\right)}+a_{\left(i+2\right)}+...+a_{\left(i+n\right)}$) = 1.0 and ($b_{i}+b_{\left(i+1\right)}+b_{\left(i+2\right)}+...+b_{\left(i+m\right)}$) = 1.0. For instance, in the case of a QoS-aware scenario, some parameters can be represented by end-to-end delay, deadline miss ratio, throughput/workload, and so on. In this specific scenario, the application level determines the relevance of each specified parameter. In the case of real-time scenarios, the most important parameter may be the deadline miss ratio of transmitted packets. It could be considered in IPI calculation with $a_{i}$ equal to 0.7. It means that IPI takes into account a deadline miss ratio with a prominence of 70%. Thus, depending on the type of application, each internal parameter can be characterized by a great or small importance (priority) in the reference context.

Moreover, regarding the $IP_{i}$ or $EP_{i}$ values, they are strictly related to the type of internal and external parameters, respectively. For instance, considering the internal parameters presented in the previous section, *Camera hardware* parameter can be identified by an appropriate value on the basis of zooming and rotation capabilities, *Event-based prioritization* parameter on the basis of relevance levels [30] and *Residual energy* parameter on the basis of the battery level. However, as mentioned before, the solution proposed in this work is independent from the number of parameters, which are defined according to application requirements.

The fuzzy control system handles the Sensing/Coding/Transmission Pattern of visual sensors taking as input crisp values of IPI and EPI, which are converted into linguistic values by using a chosen set of membership functions. The used linguistic values are:

- Very Low (VL);
- Low (L);
- Medium (M);
- High (H);
- Very High (VH).

**Figure 3:**
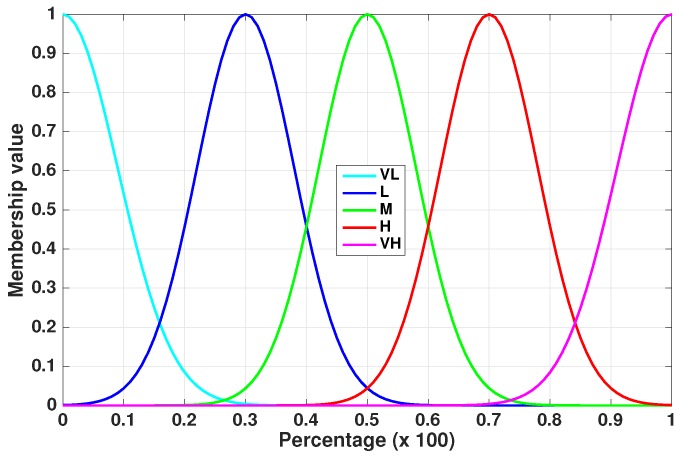
Membership functions for IPI or EPI.

The membership functions used for IPI or EPI are shown in Table 3, while their graphical representation is depicted in Figure 3.

The aim of the controller is then to elaborate on these linguistic values using an inference mechanism based on a set of if–then rules. These rules are combined in the FLC, which returns a membership function, represented, in this paper, by Gaussian functional shapes. These types of membership functions have been chosen because, as shown in [42], using Gaussian membership functions, the accuracy increases greatly, without degrading the computational performance.

Through the inference mechanism, it is possible to determine the correct output according to the fuzzy inference rules presented in Table 4. For instance, if the value of IPI is 0.48, the membership function considers the linguistic value of M, while if EPI is 0.73, the linguistic value refers to H. In this way, the final inference value is H. Finally, the conversion of this value into crisp logic decisions suitable to SCTP concludes the proposed FLC (defuzzification process).

## 5. Results

Visual sensor nodes may operate in different ways, providing information for some monitoring applications. It is clear that the sensors’ hardware parameters, the considered sensor network, the surrounding environment and existing parallel systems may influence such operations, as proposed in this work. The proposed FLC-based approach considers some parameters defined by applications and a smart city environment, presenting as results Sensing/Coding/Transmission Patterns for visual sensors.

In order to validate the proposed approach, we considered different strategies. Initially, a proof-of-concept was created assuming the case of public security in smart cities, which is a promising practical application. Then, since this article brings an innovative approach that comprises parameters that are not considered together by other works, the comparison with related works could be meaningless, which leads us to further evaluate the computation of the SCTP in different scenarios and for different internal and external parameters. At last, relevant issues when employing the proposed approach in real-world applications are discussed.

### 5.1. Computing SCTP for a Public Security Application

A hypothetical public security application was considered for initial evaluation of the proposed approach. For each considered visual sensor (or even all visual sensors equally), five different linguistic values may be computed: VL, L, M, H and VH. These values may be mapped in different configurations, according to characteristics of the designed monitoring applications.

For the WVSN considered in this example, Table 5 presents the internal and external parameters that will be taken when computing SCTP. In this particular case, we are considering three internal and three external parameters, but any number of parameters could be considered, depending on the application monitoring requirements and the desired level of integration of the wireless visual sensor network with the smart city scenario. For this WVSN, visual sensors transmit only image snapshots.

In the configuration defined at the bottom of Table 5, the FLC will indicate one of five values for each visual sensor, which will be reflected in a sensing frequency, an image resolution and an expected service for transmitted packets from the considered visual source nodes. In this case, *sensing* is defined as a frequency of snapshots taken from the monitored field, while *coding* is defined as a resolution that may be SQCIF (128 × 96), QCIF (176 × 144), SCIF (256 × 192), CIF (352 × 288) and 4CIF (704 × 576), all of them based on CIF (Common Intermediate Format) standard. At last, *transmission* is defined as an expected service, which may be transmissions with no guarantees, transmissions in a reliable way (corrupted packets are recovered) or even transmissions with reliability and timeliness assurance.

It is necessary to highlight that the relevance of the parameters depends on the characteristics of the considered applications. For the considered public security surveillance system, sensor viewing areas with low luminance should be more important. Furthermore, if sensors are battery-operated, sensors with low energy level should also be more important (since they have a shorter expected lifetime).

**Figure 4:**
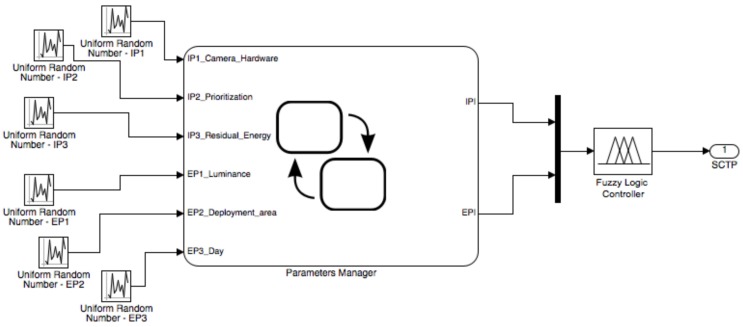
General scheme of the simulation model.

With the defined parameters for the application (top of Table 5) and adopting the FLC configuration shown in the bottom of Table 5, a configuration for the sensors can be computed. For this example, a model has been built in Simulink/Matlab (version R2015b developed by MathWorks), as shown in Figure 4. The values of $IP_{i}$ and $EP_{i}$ are acquired as input parameters of the block called *Parameters Manager*. These are random values, generated through uniform random number blocks with the ranges specified in Table 5. The Parameters Manager block manages the internal and external parameters through the Simulink/Stateflow environment, an internal Matlab tool that allows for description of the evolution of a specific system by means of a finite state machine. The output values of the Parameters Manager block are the IPI and EPI, which, subsequently, become the input parameters of the FLC.

An FLC may receive parameters reported by sensor nodes or it may exchange information with external systems. After computing the SCTP for each visual sensor, it may assign the computed information to the corresponding nodes in different ways, but we expect the adoption of application-layer protocols like the one proposed in [17].

The model depicted in Figure 4 has been implemented on the prototyping board that is shown in Figure 1. The processing unit is the Microchip PIC24FJ256GB108 microcontroller [46], which integrates the control features of a microcontroller unit with the processing and throughput capabilities of a digital signal processor. This 16-bit microcontroller has a maximum processing power of 16 MIPS (Millions of Instructions Per Second) and offers multiple serial ports (3 × I2C, 3 × SPI), 4 × UARTS and 23 independent timers. The availability of 16 kB of RAM memory for buffering, of up to 256 kB of enhanced Flash program memory and other characteristics make this microcontroller very suitable for embedded control and monitoring applications. The implementation presented here is just a proof-of-concept to show the feasibility of the proposed solution on COTS (Commercial Off-The-Shelf) devices. The obtained values are displayed on the LCD screen connected to the prototyping board. Moreover, in order to calculate the performance, the microcontroller continuously sends the output data to a computer through a serial cable.

Obviously, this implementation is valid for the case considered in this Section, which takes three internal and three external parameters. However, as this is a proof-of-concept implementation, the hardware for the FLC has to be adapted for other configurations of WVSN in smart cities, or other solutions may even be adopted for computation of SCTP, with no prejudice to the proposed approach.

Considering this generic application for public security surveillance, it is shown in Table 6 how the FLC returns the five different linguistic values for SCTP taking different combinations of IPI and EPI as inputs. In this example, $a_{1}$ and $IP_{1}$ refer to the *Camera hardware* internal parameter, $a_{2}$ and $IP_{2}$ to the *Event-based prioritization*, $a_{3}$ and $IP_{3}$ to the *Energy*, while $b_{1}$ and $EP_{1}$ refer to the *Luminance* external parameter, $b_{2}$ and $EP_{2}$ to the *Day* and, finally, $b_{3}$ and $EP_{3}$ to the *Deployment area*.

It is possible to see different combinations of IPI and EPI and their related parameters. For instance, considering the Case 5 in Table 6, the output related to the SCTP is VH. This means that the sensors will acquire two snapshots/s and the coding and the transmission configuration will be 4CIF and reliable/real-time, respectively (Table 5). It is clear that, in this case, the security surveillance application of the smart city requires more/better data. In fact, the relevance/priority of internal parameters ($a_{i}$) is 48% for *Camera hardware*, 34% for *Event-based prioritization* and 18% for *Energy*. The same remark also applies to the relevance/priority ($b_{i}$) of external parameters. As a result, the FLC processes properly the tuning of these parameters in order to return the SCTP as output. This clearly shows the adaptability of the proposed approach to various and different application contexts. In addition, the obtained results highlight that a fuzzy approach allows for ranking different parameters well and giving them a characterization for importance in the application where the FLC works. As a consequence, it is possible to obtain performance results that fit the application requirements.

### 5.2. SCTP Computation in Smart City Scenarios

The computed SCTP may typically change along with time, reflecting the dynamic nature of smart cities. In order to demonstrate such dynamism, we initially considered two distinct visual sensors in different wireless sensor networks and computed the SCTP for them. In fact, these tests are valid for generic monitoring applications in smart cities (e.g., traffic control, public security surveillance, disaster management, etc.), but different scopes could be considered [47].

**Figure 5:**
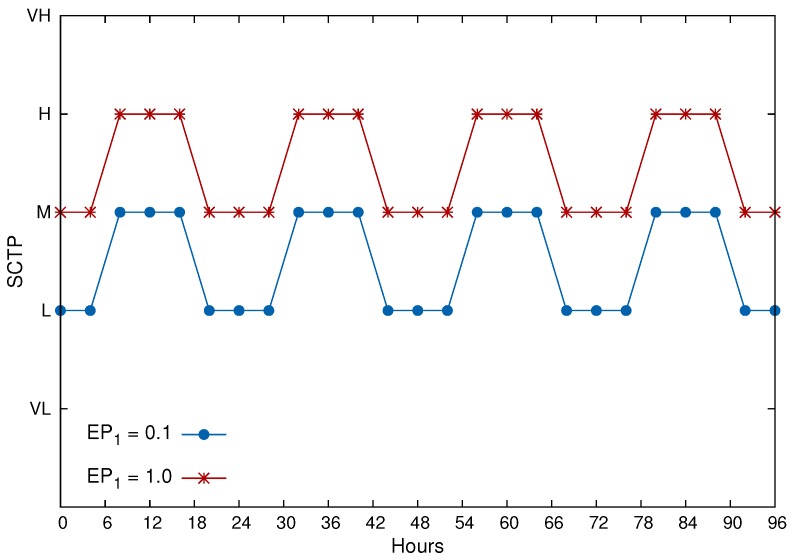
Computed SCTP for four days.

Figure 5 shows the results for computation of SCTP every 4 h for four days, from 00:00 Friday to 24:00 Monday, assuming two different WVSNs and the same configuration for all sensors of each of those wireless visual sensor networks. Three internal parameters were considered for both sensors: $IP_{1}=0.6$, $IP_{2}=0.6$ and $IP_{3}=0.6$ for $a_{1}=0.3$, $a_{2}=0.4$ and $a_{3}=0.3$. As external parameters, two of them are considered: $EP_{1}$ for deployment area and $EP_{2}$ for luminance. During the day (after 6:00 a.m. and before 6:00 p.m.), the value of $EP_{2}=1.0$, while $EP_{2}=0.1$ during the night (low luminance). Concerning $EP_{1}$, two different areas were considered, as depicted in Figure 5, for $b_{1}=0.5$ and $b_{2}=0.5$.

As can be seen in Figure 5, the SCTP will be lower during night periods and for least relevant deployment areas ($EP_{1}=0.1$), which is a very reasonable configuration for real smart city scenarios. However, as defined in the proposed approach, this is only an example, since many WVSNs may not be affected by monitoring during the night or in dark areas.

**Figure 6:**
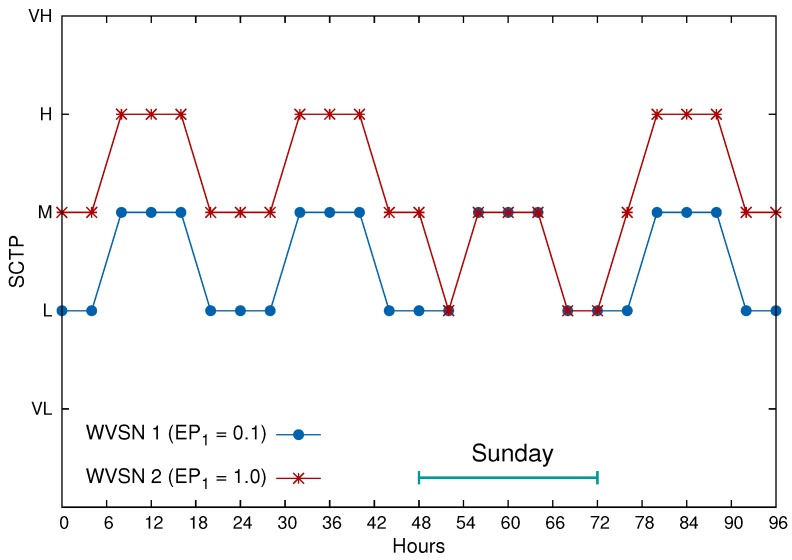
Computed SCTP for four days. Monitoring is changed on Sunday.

Figure 6 presents the values for SCTP for the same period of time, but now assumes that monitoring on Sundays is not relevant. For this, a new external parameter, $EP_{3}$, is defined. For example, in Figure 6, $EP_{3}=0.0$ for Sundays and $EP_{3}=1.0$ for the other days of the week. As a remark, the same configuration is taken from Figure 5 for WVSN 1 (only two external parameters), while WVSN 2 takes $b_{1}=0.1$, $b_{2}=0.5$ and $b_{3}=0.4$ (three external parameters).

**Figure 7:**
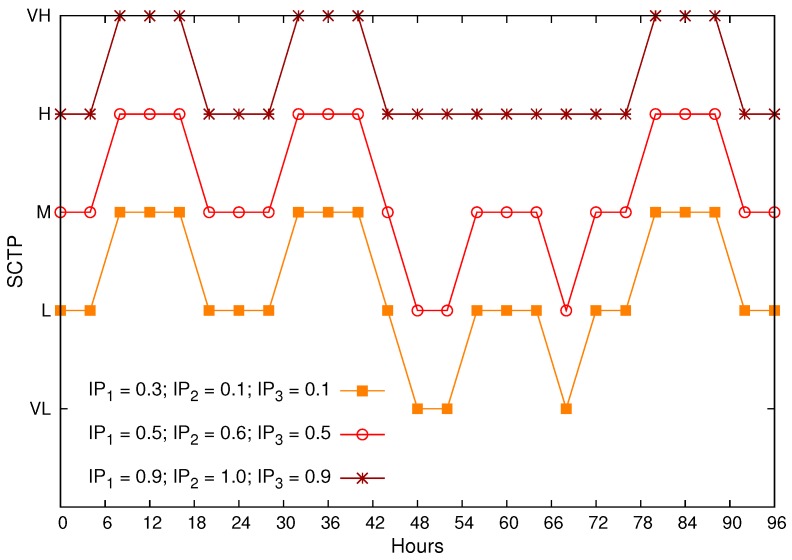
Computed SCTP for different values of internal and external parameters.

At last, for the same WVSN 2 in Figure 6, we assumed three different configurations of internal parameters, as depicted in Figure 7 for $a_{1}=0.3$, $a_{2}=0.4$ and $a_{3}=0.3$.

In Figure 7, we want to highlight how internal and external parameters are relevant when defining the SCTP, which will be reflected in sensing, coding and transmission configurations using some rules, such as the one defined in Table 5.

Actually, as sensor-based monitoring in smart cities may be highly dynamic but under a known range of possibilities (parameters may assume only known values), SCTP computation over time may be predicted and even anticipated in some cases, and this characteristic may be highly desired in smart cities with multiple concurrent WVSNs, since their sensing, coding and transmission behavior may be checked and even optimized (with specific parameters) when required.

### 5.3. Relevant Issues When Implementing the Proposed Approach

There are some relevant issues that need to be considered when implementing the proposed approach in real scenarios. Some of the most relevant ones are discussed here.

After defining the proper values for internal and external parameters, according to the nature of the wireless visual sensor network in the considered smart city, the SCTP can be dynamically computed. The next step is the configuration of the corresponding visual sensor nodes using any configuration protocol. As configuration of sensor nodes by specialized protocols is not necessarily a challenging issue, we left such implementation for future works, since it is not expected to impact the proposed approach. However, some important remarks must be made.

Although energy may not be a major concern in wireless visual sensor networks deployed in smart cities, due to the possibility to use continuous unlimited energy supplies depending on the deployment site, it is reasonable to implement energy-efficient solutions to make the overall approach flexible. In addition, concerning an SCTP assignment protocol, it may be designed having energy as an important performance parameter. In practical means, the amount of information and control messages should be kept as minimal as possible.

An SCTP assignment protocol could have a cross-layer design, exploiting the operation of other protocols [14,19], or it can be designed as an application-layer protocol. Whatever the case, the way it will operate may follow the recommendations in [17], which takes energy efficiency as the basis of the protocol used to assign sensing relevancies to source nodes. Assuming that the SCTP is computed by a single FLC, the computed SCTP can then be directed assigned to each source node using individual messages, or all information from all sensors may be broadcast through the network in combined messages. As control messages already flow in typical wireless sensor networks, SCTP assignment should not impose considerable overhead.

The way the FLC will be implemented is also a relevant issue. Actually, many FLCs may be employed in a city, uniformly or randomly distributed. This could be done, for instance, to reduce the overhead of control messages related to SCTP assignment. The size of the city and the number of concurrent wireless visual sensor networks is relevant information when making such a design choice.

Another relevant issue when employing the proposed approach is the interaction of concurrent systems. In this case, a WVSN may directly interfere in the behavior of other independent WVSN, just sending a specific control message to the FLC. For this, any WVSN may be modelled as an external parameter, which is set according to the definitions of the external sensor network. As an example, WVSN 1 modelled as an external parameter for WVSN 2 may be configured to "tune" that sensor network in the occurrence of an event that can be only detected by WVSN 1. When that event happens, WVSN 1 sends a control message to the FLC, which then takes it as an external parameter of WVSN 2. This kind of interaction may be modelled in different ways and involve any number of wireless visual sensor networks.

Finally, smart cities may be classified as having six main aspects: economy, people, governance, mobility, environment and living [47]. Each of these aspects are related to different elements of the way of life in cities, and they may be all interoperable. Actually, we expect that the proposed fuzzy-based configuration approach can be efficiently applied in any of the different scenarios of modern smart cities, since the proper configuration of visual sensors is strongly associated with the efficiency of monitoring applications.

## 6. Conclusions

Wireless visual sensor networks can be used to retrieve valuable information in an uncountable number of monitoring and control applications. However, proper configuration of sensor nodes is still a challenging task, since many parameters may influence the way sensors should operate. For higher performance, sensing, coding and transmission configuration of visual sensors should be efficiently accomplished.

An FLC-based approach was proposed in order to configure visual sensors, considering internal and external parameters of wireless visual sensor networks. We believe that this approach can benefit many WVSN applications, especially in complex scenarios like in smart city environments.

One direction for future research on the system addressed here is to improve the approach proposed in this work with a neural network able to forecast some predictable environmental parameters, i.e., to predict the monitoring conditions at different times of the day or on different days of the week. This combination would allow the fuzzy controller to make its decision taking into account not only the current situation, as detected by the sensors, but also the probable short-term evolution of the monitored environment.

## Figures and Tables

**Table 1:** Examples of different parameters for configuration of sensor nodes.

Work	Parameter	Description
[19]	Events	Events of interest are detected and used to trigger transmissions from sensor nodes, using a proposed multi-tier architecture.
[18]	Events	Scalar sensors are used to detect events of interest. Different levels of configurations of visual sensors are established based on the priority of detected events.
[20]	Events	Source nodes with higher event-based priorities transmit packets through transmission paths with lower latency.
[21]	Media type	The original media stream is split into image and audio, giving to each resulting sub-stream a particular priority when choosing transmission paths.
[22]	Node’s status	Relaying nodes may decide to drop packets according to their residual energy level and the relevance of DWT (Discrete Wavelet Transform) subbands.
[23]	Node’s status	The energy level of sensor nodes are considered when processing packets to be relayed.
[24]	Data content	The viewed segments of targets’ perimeters are associated with priority levels. Most relevant sources transmit higher quality visual data.
[25]	Network QoS	The transmission rate of source nodes is adjusted when facing congestion, silently dropping lower-relevant packets at source nodes.

**Table 2:** Range of Degradation (D) of $IP_{i}$ or $EP_{i}$ parameters.

Range of Degradation	$IP_{i}$ or $EP_{i}$ (%)
$v_{4}<D\leq v_{max}$	100→1
$v_{3}<D\leq v_{4}$	75→0.75
$v_{2}<D\leq v_{3}$	50→0.50
$v_{1}<D\leq v_{2}$	25→0.25
$v_{min}\leq D\leq v_{1}$	10→0.10

**Table 3:** Membership functions for IPI or EPI.

Linguistic Values	Interval
VL	<0.20
L	0.20÷0.40
M	0.41÷0.60
H	0.61÷0.80
VH	>0.80

**Table 4:** Inference rules of the proposed Fuzzy Logic Controller.

SCTP		IPI				
		*VL*	*L*	*M*	*H*	*VH*
EPI	*VL*	VL	VL	L	M	H
	*L*	L	L	M	M	H
	*M*	L	M	M	H	H
	*H*	M	M	H	H	VH
	*VH*	M	H	H	VH	VH

**Table 5:** FLC configuration: example of input parameters.

Parameter	D	$v_{\left(min\right)}$	$v_{\left(max\right)}$
Internal (Camera’s hardware)			
Cyclops [43]	0	0	10
MeshEye [44]	5	0	10
CMUCam [45]	10	0	10
Internal (Prioritization)			
Sensing priority	0–15	0	15
Internal (Energy)			
Energy level	0–20,000 J	20,000 J	0 J
External (Luminance)			
Luminance	10–100,000 lux	100,000 lux	10 lux
External (Day)			
Monday-Friday	10	0	10
Saturday	5	0	10
Sunday	0	0	10
External (Deployment area)			
Avenues	0	0	10
Streets	5	0	10
Public parks	8	0	10
Crowded areas	10	0	10
**SCTP**	**Sensing**	**Coding**	**Transmission**
VL	0.1 snapshot/s	SQCIF	No guarantees
L	0.2 snapshot/s	QCIF	No guarantees
M	0.5 snapshot/s	SCIF	Reliable
H	1 snapshot/s	CIF	Reliable and real-time
VH	2 snapshots/s	4CIF	Reliable and real-time

**Table 6:** Some results of the FLC.

Case	$a_{1}$	$IP_{1}$	$a_{2}$	$IP_{2}$	$a_{3}$	$IP_{3}$	$b_{1}$	$EP_{1}$	$b_{2}$	$EP_{2}$	$b_{3}$	$EP_{3}$	SCTP
1	IPI = 0.38						EPI = 0.17						VL
	0.34	0.32	0.45	0.35	0.21	0.54	0.58	0.12	0.26	0.21	0.16	0.28	
2	IPI = 0.19						EPI = 0.56						L
	0.63	0.12	0.21	0.24	0.16	0.40	0.38	0.64	0.45	0.62	0.17	0.21	
3	IPI = 0.37						EPI = 0.73						M
	0.43	0.35	0.13	0.55	0.44	0.34	0.51	0.71	0.25	0.83	0.24	0.67	
4	IPI = 0.79						EPI = 0.63						H
	0.25	0.88	0.08	0.97	0.67	0.73	0.19	0.54	0.69	0.69	0.12	0.43	
5	IPI = 0.95						EPI = 0.74						VH
	0.48	0.93	0.34	0.98	0.18	0.94	0.38	0.67	0.37	0.97	0.25	0.51	
